# Avian Influenza Risk Perception, Hong Kong

**DOI:** 10.3201/eid1105.041225

**Published:** 2005-05

**Authors:** Richard Fielding, Wendy W.T. Lam, Ella Y.Y. Ho, Tai Hing Lam, Anthony J. Hedley, Gabriel M. Leung

**Affiliations:** * University of Hong Kong, Hong Kong, Special Administrative Region, China

**Keywords:** Avian Influenza, Poultry, Exposure rate, Risk perception, Population risk, Risk behavior

## Abstract

A telephone survey of 986 Hong Kong households determined exposure and risk perception of avian influenza from live chicken sales. Householders bought 38,370,000 live chickens; 11% touched them when buying, generating 4,220,000 exposures annually; 36% (95% confidence interval [CI] 33%–39%) perceived this as risky, 9% (7%–11%) estimated >50% likelihood of resultant sickness, whereas 46% (43%–49%) said friends worried about such sickness. Recent China travel (adjusted odds ratio 0.35; CI 0.13–0.91), traditional beliefs (1.20, 1.06–1.13), willingness to change (0.29, 0.11–0.81) and believing cooking protects against avian influenza (8.66, 1.61-46.68) predicted buying. Birth in China (2.79, 1.43–5.44) or overseas (4.23, 1.43–12.53) and unemployment (3.87, 1.24–12.07) predicted touching. Age, avian influenza contagion worries, husbandry threat, avian influenza threat, and avian influenza anxiety predicted perceived sickness risk. High population exposures to live chickens and low perceived risk are potentially important health threats in avian influenza.

The risk of a pandemic human influenza strain emerging from coinfection of a human influenza carrier by avian influenza H5N1 virus is small (1); however, the potential global public health impact could be catastrophic. The circumstances that would facilitate events of low probability may be highly prevalent, which increases the public health importance of such potential events. Modern travel and transportation links would distribute a new human-transmissible influenza strain worldwide within days and overwhelm most healthcare systems within weeks. Preventing such an event is a vital public health enterprise.

Domestic waterfowl, chickens, and pigs act as aberrant hosts (2), both for avian influenza from migratory waterfowl and shorebirds (3,4) and human influenza viruses moving in the opposite species direction (2). Genetic reassortment of influenza viruses is likely more rapid in aberrant hosts because of adaptive selection pressures (5). Domestic animal and human avian influenza infection may therefore increase reassortment opportunities and the chance of a potentially pandemic strain emerging.

Most human-animal contact is domestic (pets and husbandry), or commercial, (farming, wholesale and retail marketing). Most human avian influenza infections occur among persons working or living with domesticated birds (6). Traditional Asian wet markets provide major contact points for people and live animal mixing (because of lack of refrigeration, animals are usually alive when sold), making them important potential sources of viral amplification and infection (7). Severe acute respiratory syndrome–associated coronavirus probably emerged from the selling in wet markets of Himalayan palm civets and other wild species. Traditional local demand for live animals from wet markets maintains this practice. In the wet markets of Hong Kong Special Administrative Region, ≤10 chickens are enclosed in small (approximately 25 cm × 60 cm × 60 cm) plastic cages in stacks of 5. Distressed chickens defecate, which contaminates feathers with feces. Frequent cleaning of cages and transportation and storage areas does not prevent this. Although direct hand-to-face contact is the most likely path for infection, the flapping by distressed chickens inverted during inspection by shoppers raises fecal-dust aerosols and exposes sellers, shoppers, and passers-by to any virus particles on an infected bird. Highly dense urban populations maximize opportunities for infection and transmission in any outbreak.

Minimizing unnecessary mixing between people and domestic poultry by replacing live animal sales in wet markets with hygienic central slaughtering and chilling is epidemiologically compelling. Since the 1997 Hong Kong avian influenza outbreak, which killed 6 people, all ducks and quail have been centrally slaughtered, but live chicken sales at wet markets continue, supported by chicken vaccination and intensified immunologic surveillance. The current avian influenza epidemic occurred in Asia in January 2004. By February 2, 2005, Thailand and Vietnam had documented 55 human avian influenza cases, which caused 42 deaths.

The Hong Kong government suspended imports and sales of live chickens in early 2004. Local poultry farms remain free from H5N1 infection. In May 2004, limited importation of screened live birds from China was resumed. Public consultation on central slaughtering evoked commercial and some public support for live chicken sales. To determine population knowledge of risk and self-protection practices and estimate degree of population exposure from live chicken sales, we carried out a survey in Hong Kong during February and March 2004.

## Methods

Nearly 100% of the Hong Kong population have telephones. After receiving institutional review board approval, we conducted a telephone survey of the general population from 10 a.m. to 10 p.m. from mid-February to mid-March 2004, at the peak of the avian influenza epidemic in Asia. Households were selected by using random digit dialing and, within households, respondents were selected by using random number tables based on varying household sizes. Inclusion criteria were Cantonese-Chinese speakers, age of 16 to 95 years, and residence in Hong Kong >12 months.

### Instrumentation

The draft questionnaire was examined by a panel of experts, including epidemiologists and psychologists, to determine face validity, then pilot tested with respondents, who were questioned on item comprehensibility and relevance. Interrater performance was examined by comparing response rates for recruitment and item completion, and additional training was given to improve these rates. Several households completed the questionnaire twice, with no significant differences in reported buying frequencies. Interviewers gave additional information on respondents' reactions to certain questions, which were reworked, and the instrument was retested as necessary to obtain satisfactory responses. Rater response rates were monitored throughout the study.

Of the 6-section questionnaire, 3 sections are addressed here. Section 1 consisted of Likert scale items assessing self-rated health (excellent to very poor) and influenzalike symptoms (ILI): fever, chills, cough, headache, myalgia, breathing difficulties, coryza, sore throat, diarrhea and low back pain ("yes," "no," "don't know") (8). Section 2 consisted of 13 questions on household practices of buying live birds and 3 that assessed risk perceptions: worries about catching avian influenza from buying live chickens, likelihood of self/family members getting sick from buying live chickens (all using 5- or 7-point categorical ordinal response formats) and a decile anchored 0%–100% probability assessment for likelihood of getting sick (9) from buying live chickens.

To help identify attitudinal and knowledge predictors of risk perceptions and behavior change, respondents expressed agreement or disagreement using 5-point Likert scales (strongly agree to strongly disagree) with 32 statements addressing attitudes, avian influenza protection practices, and perceptions of live chicken sales. Section 3 consisted of 9 items concerning demographic information.

### Data Analysis

Categorical data were analyzed with the χ^2^ test and continuous data with t tests. Average annual live chicken purchase rates were calculated by using a conservatively estimated number of live chicken purchases per response category. To households reporting ≤1 live chicken purchase per year, 1 live chicken purchase was attributed; to households reporting "a few times a year," 4 were attributed; to households reporting "monthly," 12 were attributed; to households reporting "a few times per month," 24 were attributed; to households reporting "weekly," 52 were attributed; and to those reporting "a few times a week," 100 purchases annually were attributed.

Perceived risk moderates behavior (10–12). To identify predictors of greater risk perception and behavior, purchase (yes/no) (model 1) and touching during purchase (yes/no) (model 2) of live chickens, and perceived likelihood of getting sick from buying live chickens (dependent variable 50th percentile dichotomized 0%–100% probability assessment responses to the question, "How likely is it that you will get sick from buying live chickens?") (model 3) were regressed in forward-stepped multivariate logistic equations on 5 attitudinal factors, adjusted for demographics. Attitudinal factors were derived by reducing the 32 attitudinal statements with varimax-rotated principal components factor analysis by using scree-plot and Eigen vector-driven factor extraction. Dichotomization and logistic regression were required for binary dependent variables in models 1 and 2 and to overcome multimodal distribution difficulties (13,14) on the response scale used in model 3. All proportions are rounded to the nearest whole number. Analyses were performed using SPSS 11.0. (SPSS Inc., Cary, NC, USA).

## Results

Seven interviewers called 6,603 telephone numbers in 4 weeks. Of these, 2,596 were invalid (fax or answering machines), and those reached at 1,765 numbers were ineligible (either non-Cantonese speakers, resident <12 months, or businesses). Of 2,240 eligible respondents, 1,256 declined to participate or complete the survey (556 were "too busy," 688 refused for other reasons), leaving 986 eligible respondents who completed the survey, a response rate of 44% (986/2,240).

The sample comprised 589 women and 397 men closely matching the most recent population census data. Men had a wider age distribution than did women (χ^2^ = 16.3, degrees of freedom [df] 5, p = 0.006), more likely to be single (χ^2^ = 23.84, df 3, p<0.001), born in Hong Kong (χ^2^ = 21.67, df 4, p<0.001), and better educated (χ^2^ = 10.52, df 3, p = 0.015) (Table 1).

**Table 1:** Sample characteristics and thematic household survey, Hong Kong, 2002

Variables	Respondents				
	No. female (%)	No. male (%)	Total (%)	Thematic household survey proportion	Effect size*
Sex	589 (60)	397 (40)	986 (100)		0.20
Male			(40)	49.8	
Female			(60)	50.2	
Age (y)†					0.16
18–34	136 (23)	111 (28)	247 (25)	30.9	
35–44	176 (30)	93 (23)	269 (27)	25.3	
45–64	215 (36)	134 (34)	349 (35)	29.7	
≥65	62 (10)	58 (15)	120 (12)	14.2	
Marital status‡					0.14
Single	108 (18)	117 (29)	225 (23)	27.1	
Married	428 (73)	262 (66)	690 (70)	63.7	
Divorced/separated	12 (2)	8 (2)	20 (2)	2.8	
Widowed	39 (7)	9 (2)	48 (5)	6.4	
Missing	2 (0)	1 (0)	3 (0)		
Place of birth§					0.11
Hong Kong	336 (57)	279 (70)	615 (62)	59.7¶	
China province	225 (38)	108 (27)	333 (34)	33.7	
Elsewhere	28 (5)	10 (2)	38 (4)	6.6	
Education#	589	397			0.24
None/primary 1–6	140 (24)	74 (18)	214 (22)	30.5	
Secondary 7–11	311 (53)	188 (48)	499 (51)	46.2	
Matriculated 12–13	32 (5)	33 (8)	65 (7)	3.6	
Tertiary	106 (18)	102 (26)	208 (21)	19.7	
Occupation	589	396			0.29
Employed	236 (41)	260 (65)	496 (51)	61.2	
Unemployed	27 (5)	45 (11)	72 (7)	5.5	
Student	28 (5)	23(6)	51 (5)	4.0	
Homemaker	254 (43)	1 (0)	255 (26)	16.3	
Retired	44 (7)	67 (17)	111 (11)	13.0	

*Three levels of effect sizes: 0.1, small; 0.3, medium; 0.5, large. †Differences between male and female participants: χ^2^= 16.30, degrees of freedom (df) = 5, p = 0.006. ‡Source: Census and Statistics Department. 2001 Population Census: Main Report. Volume I. Hong Kong: Hong Kong Government Printer; 2002. §χ^2^ = 21.67, df = 4, p<0.001. ¶ χ^2^ = 23.84, df = 3, p<0.001. # χ^2^ = 10.54, df = 3, p = 0.015. Note: Totals may not be summed to 1 due to rounding.

### Purchase of Live Chickens

One female respondent in 5 (116/589, 20%, 95% confidence interval [CI], 17%–23%) reported that her household never bought live chickens, compared to 1 in 4 (96/396, 24%, 20%–28%) male respondents who reported this. In households (78%) that reported buying live chickens, 76% (72%–78%) of female and 31% (26%–36%) of male respondents personally bought live chickens; the remainder were bought by other family members or domestic helpers. The remainder of this section only considers households that reported buying live chickens.

Of male respondents, 18% (14%–22%) reported that all family members bought live chickens, 14% (10%–18%) were the sole purchasers, while 69% (64%–74%) reported that live chickens were bought by other household members but not the respondent. The corresponding rates among females were 11% (8%–14%), 65% (61%–69%), and 24% (20%–28%). Detailed purchase patterns and rates are given in Table 2.

**Table 2:** Live chicken purchases reported by respondents

Purchasing prevalence	No. females (%)*	No. males (%)*	Multiplier†	Purchases‡	
				Female	Male
≤1/y	26 (4.4)	18 (4.5)	1	26	18
Few/y	132 (22.4)	99 (25.0)	4	528	396
»1/mo	95 (16.1)	61 (15.4)	12	1,40	732
Few/mo	112 (19.0)	60 (15.1)	24	2,688	1,440
≥1/wk	84 (14.3)	44 (11.1)	52	4,368	2,288
Few/wk	25 (4.2)	18 (4.5)	100	2,500	1,800
Subtotal§	474 (80.3)	300 (75.8)		11,250	6,674
Rate¶				23.73	22.25
Never#	116 (19.7)	96 (24.2)	0	0	
Total**	590 (100.0)	396 (100.0)			
Total average household annual purchase††				19.07	16.85
Sex-adjusted household purchase rate‡‡				18.69	

*Reported buying frequency by males and females. †Standardized number of purchases per unit time. ‡Standardized number of live chickens purchased annually (product of the proportionate buying rate [purchasing prevalence x numbers of male and female respondents buying at that rate x multiplier]). §Total annual number of live chicken purchases reported by male and female respondents (standardized). ¶Annual standardized purchase rate only among respondents reporting household purchase of live chickens (11,250/ 474 [female], 6,674/300 [male]). #Proportion reporting their household never buys live chickens. **Total households in sample. ††Total reported household annual purchases (b)/number of women (590) and men (396) buying live chickens (subtotal). ‡‡In households buying live chickens, 14% of male respondents and 65% of female respondents make purchases. We have therefore assumed that the remaining purchases noted by 86% of male respondents within buying households are made by women at the higher female rate. We assumed that of the remaining 35% of female respondent households, 14% of purchases would be by men (at the male rate) and the remainder by women. The resulting figure is the overall sex-proportionately adjusted buying rate and is used as the estimated average household buying rate.

Because 65% of women but only 14% of men personally bought live chickens, we adjusted for sex differences in purchasing rates by applying the female rate to the remaining proportion of purchases in male-respondent households (86%), and all but 14% in female respondent households, the remainder attributed at the male rate. The sex-adjusted household purchase rate for all 2,051,890 households in Hong Kong is given in Table 2.

### Contact with Live Chickens during Purchase

Of the 78% of respondents who reported their household bought live chickens, 13% (10%–16%) of female and 19% (14%–23%) of male purchasers touched chickens when buying. Overall, 14% (9%–13%) of purchases involved physical contact with a live chicken. Extrapolating these exposures (14% of 78% = 11%) by the average number of chickens purchased annually (18.7), multiplied by the number of Hong Kong households (2,051,890), gives 4,220,738 person-chicken exposures annually. Of those reporting that they touched live chickens when buying, only ≈30% said they "always" or "usually" wash hands afterwards. Anxiety scores did not differ between those who bought live chickens and those who did not.

### Risk Perception

This section addressed all respondents, not just those buying live chickens. Four separate items tapped perception of risk from buying live chickens. The first assessed perceived "objective" risk. Overall, 36% (33%–39%) of respondents agreed with the statement "Buying live chickens is risky to health." The next 2 items considered perceived consequences of risk (odds of getting sick). Statement-based probability estimates for "getting sick from buying live chickens" indicated that 34% (31%–37%) of respondents considered that they would "never" or were "very unlikely" to get sick from buying live chickens, while 27% (24%–30%) thought it was "unlikely," 24% (21%–27%) "chances are even" and 15% (13%–17%) "likely" or "very likely." The third item (0%–100% probability estimates of sickness risk) produced lower risk estimates than the second item, with 53% (50%–56%) perceiving the likelihood of getting sick at below 26%, 38% (35%–41%) in the range 26%–50%, and 9% (7%–11%), exceeding a 51% likelihood. Item 4 assessed the risk expressed by others. Overall, 46% (95% CI 43%–49%) of respondents reported that their friends had expressed worries about catching avian influenza. Risk perceptions did not differ by age, sex, education, income, or occupation.

### Factor Analysis

The 32 attitude statements produced a 5-factor best-fit solution, which accounted for 38.5% of the score variance (Table A1). These 5 factors were labeled according to their item content. Factor 1, "animal husbandry risk" (10% of variance), included items attributing avian influenza to market practices, live animal sales, and poor home and market hygiene. Factor 2, "traditional market practices" (9% of variance), items supported traditional markets, their low health risks, live chicken sales, and trivialized health "scares." Factor 3, "protective practice" (8%), items reflected unwillingness to continue live chicken purchases despite risks, unwillingness to take risks for enjoyment, risks from zoonotic infections, and responsibility for own health. Factor 4, "avian influenza anxieties" (6%), items reflected avian influenza worries, effect of media reports, and sense of vulnerability. Factor 5, "feel protected" (6%), items reflected reassurance from media reports, trust in government, and confidence in existing avian influenza control measures.

Multivariate logistic models 1–3 were adjusted for sex, age, marital status, education, occupation, income, place of birth, years of residence in Hong Kong, and recent China travel (Table A1). All models also included factors 1–5 plus attitudinal items not included in the factor scores.

Model 1 produced 6 independent predictors of buying live chickens: 1) travel; respondents reporting recent Chinese mainland travel were less likely to buy (adjusted odds ratio [AOR] 0.35, 95% CI 0.1–0.9); 2) employment status; unemployed people were less likely to buy (AOR 0.18, 0.05–0.6); 3) traditional market practices (Factor 2 score); persons supporting traditional markets were more likely to buy (AOR 1.2, 1.06–1.1); 4) protective practice (Factor 3 score); persons reporting high protective practices were more likely to buy (AOR 1.2, 1.6–1.5); 5) willingness to change buying habits if other persons do the same (AOR 0.3, 0.1–0.8); and 6) belief that cooking food thoroughly is the best protection against bird flu (AOR 8.7, 1.6–46.7).

Model 2 estimated independent predictors of touching chickens when buying, using only respondents who reported buying live chickens themselves (n = 451). Two variables independently predicted higher risk of touching: place of birth; persons born outside of Hong Kong (AOR [China] 2.8, 1.4–5.4; [elsewhere] 4.2, 1.4–12.5), and employment status; unemployment (AOR 3.9, 1.2–12.1).

Model 3 identified adjusted independent predictors of risk perceptions for getting sick from buying live chickens. Older age lowered perceived risk (AOR for those ≥54 years of age 0.3, 0.2–0.6; 35–54 years 0.5, 0.3–0.8 [reference 18–34 years]), while worries about catching bird flu (AOR 2.9, 1.9–4.5), animal husbandry risk (Factor 1) (AOR 1.1, 1.04–1.14), protective practices (Factor 3) (AOR 1.1, 1.04–1.2), and avian influenza anxiety (Factor 4) (AOR 1.1, 1.0–1.2), all increased risk perception.

## Discussion

Women are usually responsible for food shopping; shopping practices differ by sex, and reporting differences by sex are found elsewhere (15). The observed purchase (and therefore exposure) rate of 18.7 live chickens/household/year (38,370,343 purchases annually) matches government figures of ≈38,325,000 live chickens purchased annually in Hong Kong, (Government of Hong Kong, 2004). This provides important independent validation of our data accuracy.

How much risk this exposure represents is difficult to accurately quantify. A highly conservative estimation assumes that genetic reassortment of human and avian influenza viruses can occur only on day l of a 5-day infectious period (16) in a person with human influenza. During the two 10-week human influenza seasons that occur annually in Hong Kong (17,18), sentinel data for ILI 1998–2004 indicate that peak population infection rates (p_i_) average 10% (±50% lower and upper bound estimates, i.e., 5%-15%), giving 0.2 × (4,220,738/52) × 20 × p_i_ = 32,467 (16,233–48,700) episodes when persons on day 1 of a human influenza infection face exposure to live chickens. Wet markets amplify viral loads (19). Before the enactment in 2003 of wet market "rest days," H5N1 isolates occurred in ≈10% of chickens for sale in Hong Kong (20). Because all live chickens available in Hong Kong are vaccinated against avian influenza and the vaccine is presumed 90% effective (1,21), then only 1% (10% of 10% carrier rate) are potentially avian influenza infected, giving 325 (162–487) day 1 potential coinfection exposures when reassortment could occur, a rate of 0.0077% (0.0038–0.0115%). Influenza produces no symptoms for 24 to 48 hours after infection so shopping rates would be unaffected. Assuming that 50% of persons shop on day 1 of infection reduces the figure by half to 162 (81–243) coinfection exposures annually. Among the 11% who touch the chickens, risk for avian influenza infection is likely greater. These estimates, though highly uncertain, quantify the potential risk magnitudes involved.

These 4.2 million exposures provide substantial opportunity for chicken-to-human transmission in Hong Kong wet markets. Elsewhere in Asia, exposure events are likely even more common for 2 reasons. First, persons born outside Hong Kong and China touch chickens more frequently. Second, most other Southeast Asian countries have endemic avian influenza infections and have not implemented intense surveillance, widespread inoculation of imported chickens, or both, or monthly market rest days to reduce viral load in markets.

Although one third of respondents perceived some risks from live chicken sales, risk magnitude seldom exceeded 60%, and peaks at 25% and 50% are partially artifactual (13,14). Almost 50% indicated that their friends had expressed anxieties about avian influenza. Attributing greater concerns to others than to oneself reflects optimistic attribution bias, a protective response enabling expression of concern while preserving "face" (22). Sickness anxieties reflected the fact that the markets and live chicken sales were perceived as health threats. Older persons, possibly due to past experience of buying live chickens, or past "chicken plagues," viewed the present avian influenza outbreak as low risk. Hazard familiarity and experience reduce associated risk perceptions (23). Yet respondents who reported higher anxiety and greater risk were no less likely to buy live chickens.

Raising population anxiety levels by warnings about disease produces only transient, inconsistent, and therefore often ineffective results as a means of reducing long-term high-risk behavior for 3 main reasons. First, persons perceiving control over dubious "hazards" may underestimate the associated risk, which reduces the likelihood of behavior change (24). Second, persons who perceive little or no control over a threat adopt fatalistic responses, continue with established behavior, and direct coping efforts towards controlling emotions rather than risks (25,26). Third, hazard exposure causes familiarity, thus reducing perceptions of risk (10–12,27). Therefore, persons may dismiss the warnings as exaggerated or unrealistic.

However, some persons are willing to change buying habits if others do. Consequently, health warnings can produce short-term effects that rapidly attenuate in the absence of increased perceived threat, particularly where established behavior is involved. This suggests that large group changes may be more probable than individual level changes, consistent with evidence from health "scares." Once confidence in food safety is lost, recovery time may be protracted (28).

In conclusion, perceptions of risk from buying live chickens were moderate, but sickness anxieties did not predict buying or touching habits. Buying was, importantly, strongly predicted by the erroneous belief that cooking is the best way to protect from avian influenza. This is an important message for health education groups seeking to increase preventive practices to control possible avian influenza outbreaks.

We received financial support from the Research Fund for the Control of Infectious Diseases of the Health, Welfare, and Food Bureau of the Hong Kong Special Administrative Region Government.

## Appendix

**Table A1:** Factor structure of attitude and knowledge items*

Variable	Factor structure†				
	Factor 1: animal husbandry risk	Factor 2: traditional market practices	Factor 3: risk taking	Factor 4: AI anxieties	Factor 5: feel protected
SARS due to market hygiene	0.693				
AI due to market hygiene	0.722				
SARS and AI due to unsustainable animal husbandry practices	0.626				
Live animal sales important disease risk	0.533				
SARS and AI due to market demand for live animals	0.621				
SARS and AI due to poor personal hygiene	0.468				
Raising and selling live chickens should be prohibited		–0.561			
Buying live chickens risky to health		–0.446			
Do not buy live chickens, despite what others do		–0.429			
Defend the right to trade live animals, even at risks to community's health		0.580			
Occasional health scares should not stop tradition of buying live chicken		0.629			
Stopping live poultry sales makes people lose livelihood		0.640			
Risk health for taste of live chicken			–0.527		
Need more info on AI			0.501		
Risk health for benefit of buying live chickens			–0.615		
I'm responsible for protecting myself from AI			0.513		
Believe people who say animal diseases nothing to worry about			–0551		
Friends worry about AI				0.564	
AI news in papers scares me				0.646	
Feel vulnerable to AI				0.554	
Scared of catching AI				0.505	
TV/radio AI news reassuring					0.418
Trust government to protect health					0.679
Actions implemented to control AI are inadequate					0.705

*SARS, severe acute respiratory syndrome; AI, avian influenza. †Varimax rotation, all factor loadings <0.4 suppressed.
